# Clinical predictive value of pre-pregnancy tests for unexplained recurrent spontaneous abortion: a retrospective study

**DOI:** 10.3389/fmed.2024.1443056

**Published:** 2024-08-07

**Authors:** Jinming Wang, Dan Li, Zhenglong Guo, Yanxin Ren, Li Wang, Yuehua Liu, Kai Kang, Weili Shi, Jianmei Huang, Shixiu Liao, Yibin Hao

**Affiliations:** ^1^Medical Genetics Institute of Henan Province, Henan Provincial People’s Hospital, Zhengzhou University People’s Hospital, Zhengzhou, China; ^2^National Health Commission Key Laboratory of Birth Defects Prevention, Henan Key Laboratory of Population Defects Prevention, Zhengzhou, China; ^3^Institute of Information Technology, PLA Strategic Support Force Information Engineering University, Zhengzhou, China; ^4^Obstetrics Department of The Third Affiliated Hospital of Zhengzhou University, Zhengzhou, China; ^5^Institute for the Prevention and Treatment of Chronic Noncommunicable Diseases, Henan Provincial Center for Disease Control and Prevention, Zhengzhou, China

**Keywords:** recurrent spontaneous abortion, risk prediction model, pre-pregnancy tests, logistic regression analysis, retrospective study

## Abstract

**Introduction:**

Early prediction and intervention are crucial for the prognosis of unexplained recurrent spontaneous abortion (uRSA). The main purpose of this study is to establish a risk prediction model for uRSA based on routine pre-pregnancy tests, in order to provide clinical physicians with indications of whether the patients are at high risk.

**Methods:**

This was a retrospective study conducted at the Prenatal Diagnosis Center of Henan Provincial People’s Hospital between January 2019 and December 2022. Twelve routine pre-pregnancy tests and four basic personal information characteristics were collected. Pre-pregnancy tests include thyroid-stimulating hormone (TSH), free triiodothyronine (FT3), free thyroxine thyroid (FT4), thyroxine (TT4), total triiodothyronine (TT3), peroxidase antibody (TPO-Ab), thyroid globulin antibody (TG-Ab), 25-hydroxyvitamin D [25-(OH) D], ferritin (Ferr), Homocysteine (Hcy), vitamin B12 (VitB12), folic acid (FA). Basic personal information characteristics include age, body mass index (BMI), smoking history and drinking history. Logistic regression analysis was used to establish a risk prediction model, and receiver operating characteristic (ROC) curve and decision curve analysis (DCA) were employed to evaluate the performance of prediction model.

**Results:**

A total of 140 patients in uRSA group and 152 women in the control group were randomly split into a training set (*n* = 186) and a testing set (*n* = 106). Chi-square test results for each single characteristic indicated that, FT3 (*p* = 0.018), FT4 (*p* = 0.048), 25-(OH) D (*p* = 0.013) and FA (*p* = 0.044) were closely related to RSA. TG-Ab and TPO-Ab were also important characteristics according to clinical experience, so we established a risk prediction model for RSA based on the above six characteristics using logistic regression analysis. The prediction accuracy of the model on the testing set was 74.53%, and the area under ROC curve was 0.710. DCA curve indicated that the model had good clinical value.

**Conclusion:**

Pre-pregnancy tests such as FT3, FT4, TG-Ab, 25-(OH)D and FA were closely related to uRSA. This study successfully established a risk prediction model for RSA based on routine pre-pregnancy tests.

## Introduction

Recurrent Spontaneous Abortion (RSA) is one of the most common pregnancy complications in obstetrics and gynecology (1, 2). The incidence rate of RSA is about 2% (2, 3). RSA Patients usually have experienced multiple miscarriages before diagnosis. RSA is not only a serious threat to the physical and mental health of patients, but also a heavy economic burden to patients and their families (1, 2, 4). Due to the complex and diverse etiology as well as the lack of specific clinical manifestations in patients, the standardized diagnosis and treatment of RSA have become an important issue that urgently needs to be addressed in the field of reproductive health (2, 5).

The international definition of RSA is not uniform. The Royal College of Obstetricians and Gynecologists (RCOG) defines RSA as three or more pregnancy loss before 24 weeks of pregnancy (6). The European Society of Human Reproduction and Embryology (ESHRE) defines RSA as two or more pregnancy loss before 24 weeks of pregnancy (7). The German Society of Gynecology and Obstetrics (DGGG), the Austrian Society of Gynecology and Obstetrics (OGGG) and the Swiss Society of Gynecology and Obstetrics (SGGG) define RSA as three or more consecutive pregnancy loss before 20 weeks of pregnancy (8). Chinese Society of Obstetrics and Gynecology. The Chinese Society of Obstetrics and Gynecology recommends to define RSA as two or more consecutive pregnancy loss with the same spouse before 28 weeks of pregnancy, which was uses as inclusion criteria for uRSA group in this paper (9).

The currently known causes of RSA include the following aspects (1, 10). Chromosomal abnormalities in embryos are one of the most common causes of RSA. About 4 to 8% of couples experiencing RSA have chromosomal abnormalities. RSA is also associated with abnormal uterine anatomy, such as including mediastinal uterus, unicornuate uterus and bicornuate uterus. Common endocrine factors such as luteal insufficiency, multiple ovarian syndromes, hyperprolactinemia and abnormal glucose metabolism can increase the risk of RSA. Common autoimmune diseases, such as antiphospholipid syndrome, systemic lupus erythematosus and undifferentiated connective tissue disease, are also associated with the occurrence of RSA. The pre-thrombotic state of pregnant women can affect the blood circulation of the uterus and placenta, and can easily lead to miscarriage or premature birth. Recent studies have shown that an imbalanced microenvironment at the maternal fetal interface and infection with reproductive tract pathogens may also lead to RSA. However, the specific causes and pathogenesis of nearly 40% of recurrent miscarriages are still unclear, which is called unexplained recurrent spontaneous abortion (uRSA) (11).

In clinical diagnosis and treatment, the screening indicators of RSA involve multiple personalized examination items such as chromosome karyotype analysis and thromboela-stogram, which are often used for etiological analysis after the diagnosis of recurrent miscarriage. It is difficult to conduct effective early intervention before diagnosis of uRSA (1, 4). Thyroid function indicators, 25-hydroxyvitamin D [(25-(OH)) D], ferritin (Ferr), homocysteine (Hcy), vitamin B12 (VitB12) and folic acid (FA) are routine test items for pre-pregnancy women (12–15). Previous studies have shown that some of these factors may be related to uRSA, but the clinical reliability and accuracy of predicting the risk of uRSA based on a single indicator are difficult to guarantee (15–17).

Early prediction and intervention are crucial for the prognosis of uRSA. The main purpose of this study is to establish a risk prediction model for uRSA based on routine pre-pregnancy tests, in order to provide clinical physicians with indications of whether the patients are at high risk. To our knowledge, this study is the first time to use logistic regression to establish a risk prediction model for uRSA based on pre-pregnancy tests. This study has important value for early intervention and improving prognosis in patients with uRSA.

## Materials and methods

### Study design

This was a retrospective study conducted at the Prenatal Diagnosis Center of Henan Provincial People’s Hospital between January 2019 and December 2022. In this study, the Chinese definition of RSA was adopted and 8 strict exclusion criteria were formulated. Twelve pre-pregnancy tests and four basic personal information characteristics were collected through blood tests, clinical medical records or follow-up questionnaires before the first pregnancy loss. All participants who did not conform to the inclusion criteria, who conformed to the exclusion criteria, or who had incomplete test data were excluded.

### Patients’ selection

**Figure 1:**
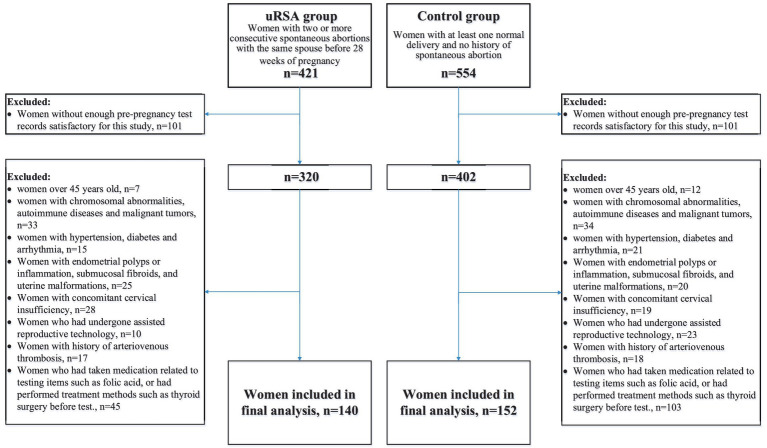
Flowchart of participant inclusion process.

The participants in this study were women who underwent preconception counseling. We set up an uRSA group and a control group. According to Chinese expert consensus on diagnosis and management of recurrent spontaneous abortion, the inclusion criteria for uRSA group was the occurrence of two or more consecutive spontaneous abortions with the same spouse before 28 weeks of pregnancy (9). The inclusion criteria for control group were the occurrence of at least one normal delivery with no history of spontaneous abortion. To reduce confounding caused by known etiology, drugs and treatments related to pre-pregnancy tests, we formulated 8 exclusion criteria: (1) women over 45 years old, (2) women with chromosomal abnormalities, autoimmune diseases and malignant tumors, (3) women with hypertension, diabetes and arrhythmia, (4) women with endometrial polyps or inflammation, submucosal fibroids, and uterine malformations, (5) women with concomitant cervical insufficiency, (6) women who had undergone assisted reproductive technology, (7) women with a history of arteriovenous thrombosis, (8) women who had taken medication related to test items such as folic acid, or had performed treatment methods such as thyroid surgery before test. Figure 1 showed the flowchart of participant inclusion process.

### Main outcome measures

Twelve pre-pregnancy tests and four basic personal information characteristics of all patients were collected. Pre-pregnancy tests included thyroid-stimulating hormone (TSH), free triiodothyronine (FT3), free thyroxine thyroid (FT4), thyroxine (TT4), total triiodothyronine (TT3), peroxidase antibody (TPO-Ab), thyroid globulin antibody (TG-Ab), 25-hydroxyvitamin D [25-(OH) D], ferritin (Ferr), homocysteine (Hcy), vitamin B12 (VitB12), folic acid (FA). The pre-pregnancy test items selected in this study were mainly considered for two reasons: Firstly, every women who received pregnancy consultation at Prenatal Diagnosis Center of Henan Provincial People’s Hospital was suggested to undergo the above test items. Secondly, studies have shown that these items may be related to uRSA. The reference ranges of the above pre-pregnancy tests in Henan Provincial People’s Hospital were shown in Table 1. Basic personal information characteristics included age, body mass index (BMI), smoking history and drinking history. Pre-pregnancy tests were obtained through blood tests, and basic personal information characteristics were obtained through clinical medical records or follow-up questionnaires. Due to the goal of predicting the risk of uRSA in this study, all the information and indicators were collected within 6 months prior to the first pregnancy which leads to miscarriage in the uRSA group, and all the information and indicators were collected within 6 months prior to the first pregnancy in the control group.

**Table 1:** Reference ranges of pre-pregnancy tests.

Pre-pregnancy tests	Reference ranges
Thyroid-stimulating hormone (TSH)	0.35 ~ 4.94 uIU/mL
Free triiodothyronine (FT3)	2.43 ~ 6.01 pmol/mL
Free thyroxine thyroid (FT4)	9.01 ~ 19.05 pmol/mL
Thyroxine (TT4)	62.68 ~ 150.84 nmol/mL
Total triiodothyronine (TT3)	0.54 ~ 2.96 nmol/mL
Peroxidase antibody (TPO-Ab)	0 ~ 34.00 IU/mL
Thyroid globulin antibody (TG-Ab)	0 ~ 115.00 IU/mL
25-hydroxyvitamin D [(25-(OH) D)]	30.00 ~ 100.00 ng/mL
Ferritin (Ferr)	4.63 ~ 204.00 ng/mL
Homocysteine (Hcy)	5.08–15.39 μmol/mL
Vitamin B12 (vitb12)	187.00–883.00 pg./mL
Folic acid (FA)	3.10 ~ 20.50 ng/mL

### Statistical analysis

For missing values in the data, multiple imputation method was used for processing (18). In order to improve the convergence performance of the model, we selected the optimal cutoff value based on X-tile tool and converted continuous variables into categorical variables. X-tile is a bioinformatics tool for biomarker assessment and cut-point optimization (19). Chi square test was used to analyze the significance of differences in various characteristics between uRSA patients and the control group, and *p* < 0.05 was considered to be statistically significant. The dataset with selected characteristics was randomly split into two distinct subsets: a training set (*n* = 186) to construct the model and a testing set (*n* = 106) to test the model. Logistic regression analysis was used to establish a risk prediction model, and receiver operating characteristic (ROC) curve and decision curve analysis (DCA) were employed to evaluate the performance of prediction model. All statistical analyses were performed with the use of Statistical Product and Service Solutions (SPSS) version 27.

## Results

### Significance analysis of differences between uRSA group and control group

After rigorous screening, a total of 140 patients in uRSA group and 152 women in the control group were incorporated into this study. For determining risk factors that cause uRSA, optimal cutoff value calculated by X-tile tool and chi-square test results for each single characteristic between uRSA group and control group were presented in Table 2. There was no significant difference in all basic personal information characteristics. Only FT3 (*p* = 0.018), FT4 (*p* = 0.048), 25-(OH) D (*p* = 0.013) and FA (*p* = 0.044) were closely related to uRSA among pre-pregnancy tests.

**Table 2:** Chi-square test for each single factor based on all participants (*n* = 292).

Characteristics	Control group (*n* = 152)	uRSA group (*n* = 140)	χ^2^	*p*-value
**Age**				
<32	80 (52.63%)	92 (65.71%)	2.576	0.108
≥32	72 (47.37%)	48 (34.29%)		
**Body mass index**				
<21.62	70 (46.05%)	68 (48.57%)	0.093	0.761
≥21.62	82 (53.95%)	72 (51.43%)		
Smoking history*				
No	142 (93.42%)	120 (85.71%)	2.348	0.125
Yes	10 (6.58%)	20 (14.29%)		
Drinking history*				
No	136 (89.47%)	112 (80.00%)	2.555	0.110
Yes	16 (10.53%)	28 (20.00%)		
**Thyroid-stimulating hormone**				
<1.17 uIU/mL	30 (19.74%)	32 (22.86%)	0.023	0.989
≥1.17 uIU/mL	122 (80.26%)	108 (77.14%)		
**Free triiodothyronine**				
<3.80 pmol/mL	26 (17.11%)	4 (2.86%)	8.081	0.018
3.80 ~ 4.13 pmol/mL	30 (19.74%)	30 (21.43%)		
≥4.13 pmol/mL	96 (63.15%)	106 (75.71%)		
**Free thyroxine thyroid**				
<12.03 pmol/mL	10 (6.58%)	28 (20.00%)	6.054	0.048
12.03 ~ 15.44 pmol/mL	126 (82.89%)	96 (68.57%)		
≥15.44 pmol/mL	16 (10.53%)	16 (11.43%)		
**Thyroxine**				
<125.74 nmol/L	136 (89.47%)	118 (84.29%)	0.866	0.352
≥125.74 nmol/L	16 (10.53%)	22 (15.71%)		
**Total triiodothyronine**				
<1.75 nmol/L	140 (93.33%)	118 (84.29%)	2.166	0.141
≥1.75 nmol/L	12 (6.67%)	22 (15.71%)		
**Peroxidase antibody**				
<12.00 IU/mL	136 (89.47%)	114 (81.43%)	1.915	0.166
≥12.00 IU/mL	16 (10.53%)	26 (18.57%)		
**Thyroid globulin antibody**				
<11.46 IU/mL	128 (84.21%)	102 (72.86%)	2.808	0.094
≥11.46 IU/mL	24 (15.79%)	38 (27.14%)		
**25-hydroxyvitamin D**				
<11.60 ng/mL	46 (30.26%)	76 (46.51%)	8.651	0.013
11.60 ~ 14.00 ng/mL	44 (28.95%)	26 (39.53%)		
≥14.00 ng/mL	62 (40.79%)	38 (13.96%)		
**Ferritin**				
<28.49 ng/mL	62 (40.79%)	60 (42.86%)	0.064	0.800
≥28.49 ng/mL	90 (59.21%)	80 (57.14%)		
**Homocysteine**				
<9.18 μmol/mL	116 (76.32%)	102 (72.86%)	0.230	0.631
≥9.18 μmol /mL	36 (23.68%)	38 (27.14%)		
**Vitamin B12**				
<305 pg./mL	20 (13.16%)	22 (15.71%)	0.193	0.660
≥305 pg./mL	132 (86.84%)	118 (84.29%)		
**Folic acid**				
<5.40 ng/mL	8 (5.26%)	20 (14.29%)	6.249	0.044
5.40 ~ 15.50 ng/mL	102 (67.11%)	100 (71.42%)		
≥15.50 ng/mL	42 (27.63%)	20 (14.29%)		

*Smoking history refers to smoking at least one cigarette per day for at least six months. Drinking history refers to drinking alcohol at least once a week and consuming ethanol more than 20 g.

### Prediction model using logistic regression

**Figure 2:**
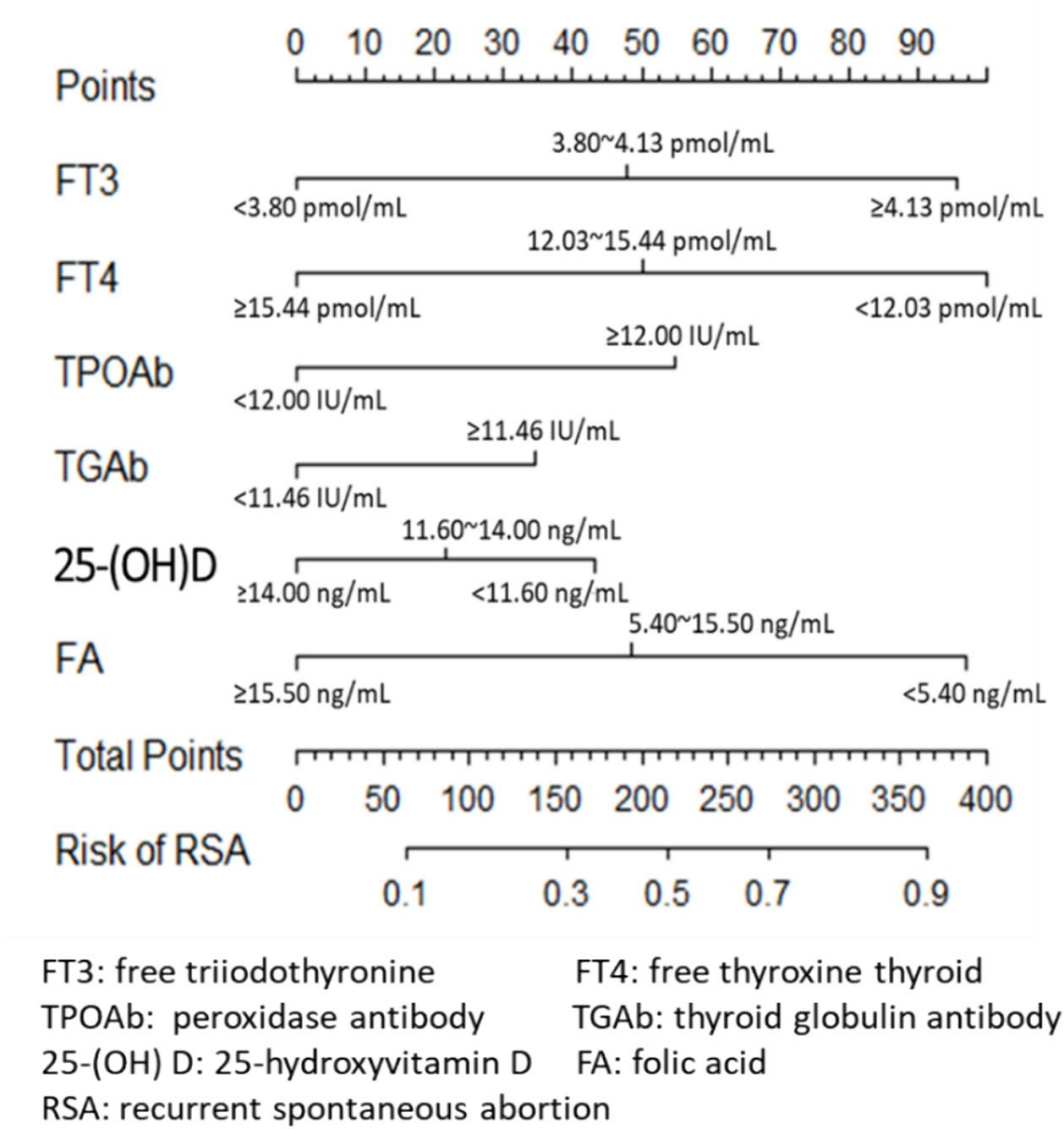
Nomogram of logistic regression model.

Clinical experience and previous studies suggest that TPO-Ab and TG-Ab are important influencing factors for the occurrence of uRSA and may have a mixed effect with other indicators such as FT3 and FT4. The logistic regression model was established using the training set with FT3, FT4, 25-(OH) D, FA, TPO-Ab and TG-Ab. The results obtained by the established model were given in Table 3, and the nomogram was given in Figure 2. According to the contribution of each influencing factor in the model to the outcome variable (the size of the regression coefficient), the nomogram assigns a score to each value level of each influencing factor. We could intuitively see that FT3, FT4 and FA may have a greater impact on uRSA than the other characteristics.

**Table 3:** Results of logistic regression model based on the training set (*n* = 186).

Characteristics	OR	95%CI	*p*-value
**Free triiodothyronine**			
<3.80 pmol/mL	1.000		
3.80 ~ 4.13 pmol/mL	7.916	1.492–42.000	0.019
≥4.13 pmol/mL	0.854	0.337–2.161	0.738
**Free thyroxine thyroid**			
<12.03 pmol/mL	1.000		
12.03 ~ 15.44 pmol/mL	0.219	0.044–1.075	0.061
≥15.44 pmol/mL	1.049	0.321–3.428	0.937
**Peroxidase antibody**			
<12.00 IU/mL	1.000		
≥12.00 IU/mL	0.307	0.066–1.426	0.132
**Thyroid globulin antibody**			
<11.46 IU/mL	1.000		
≥11.46 IU/mL	0.778	0.235–2.572	0.681
**25-hydroxyvitamin D**			
<11.60 ng/mL	1.000		
11.60 ~ 14.00 ng/mL	0.544	0.213–1.388	0.203
≥14.00 ng/mL	1.485	0.506–4.358	0.472
**Folic acid**			
<5.40 ng/mL	1.000		
5.40 ~ 15.50 ng/mL	0.157	0.032–0.785	0.024
≥15.50 ng/mL	0.391	0.134–1.145	0.087

**Figure 3:**
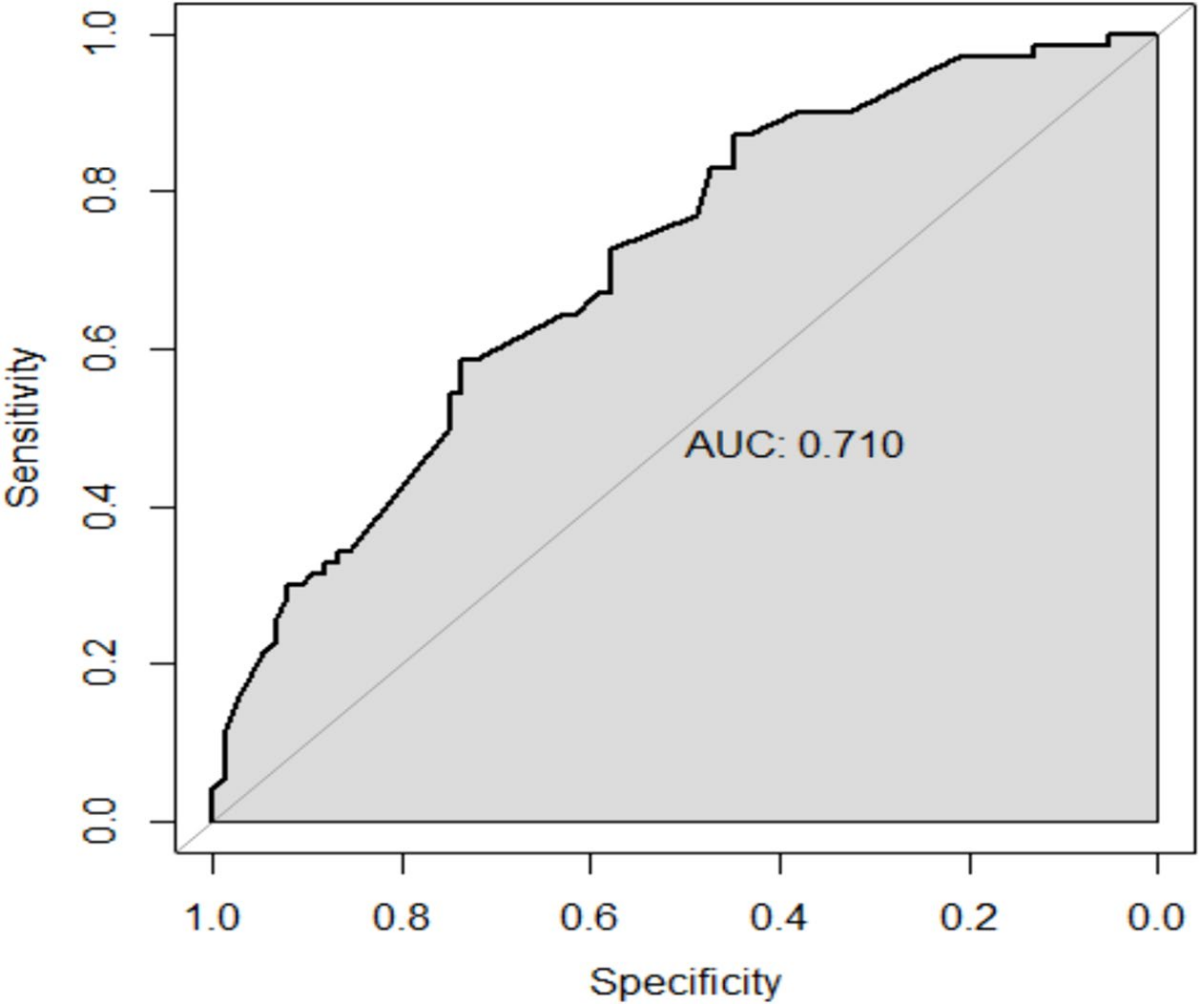
ROC curve of logistic regression model.

**Figure 4:**
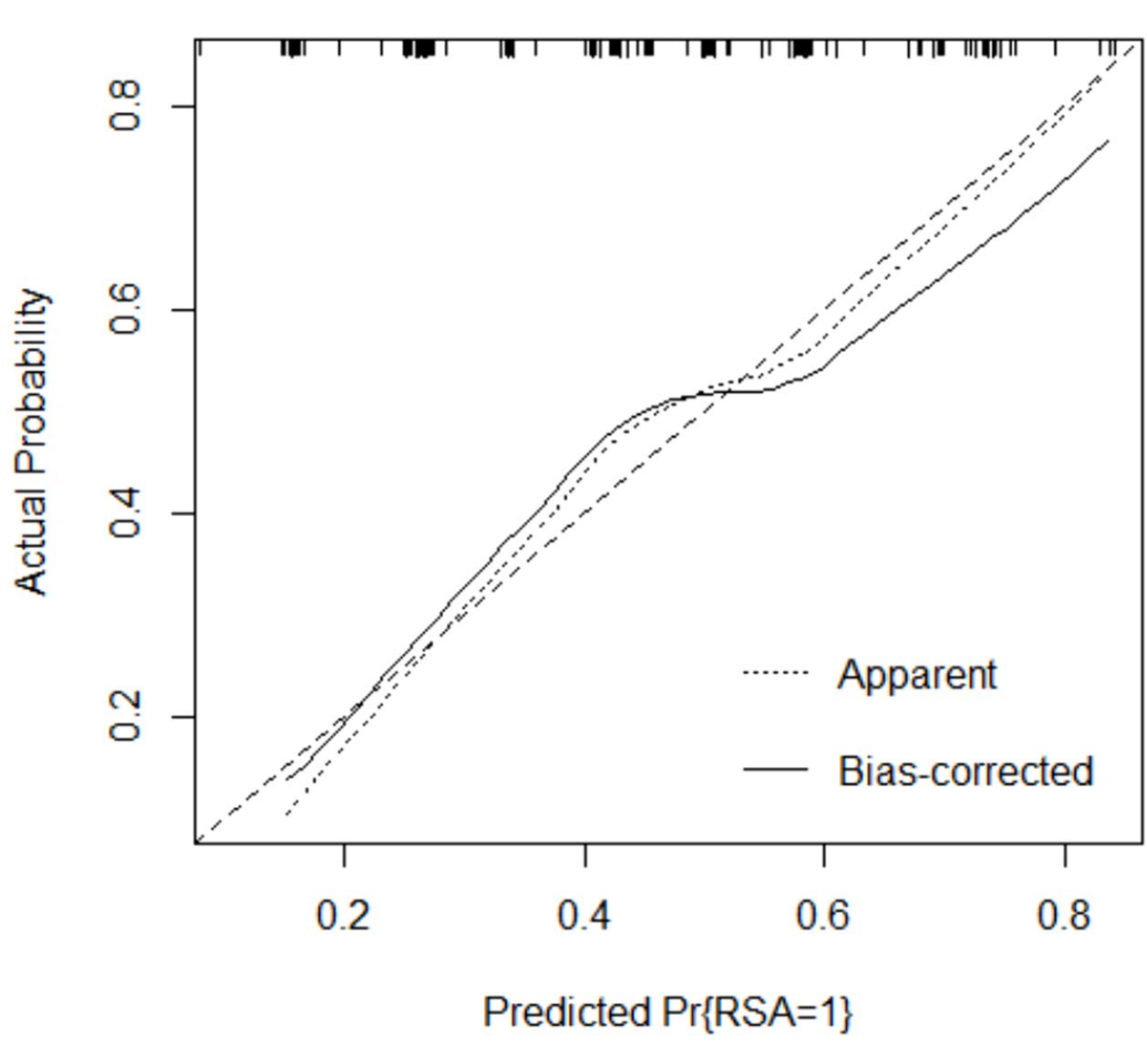
Calibration plot of logistic regression model.

The performance of the model was validated using the testing set. The accuracy, sensitivity, and specificity of the testing set were 74.53, 76.47, and 72.73% as shown in Table 4. The receiver operating characteristic (ROC) curve was shown in Figure 3 and the area under curve (AUC) was 0.710 (95% CI, 0.621–0.799). The calibration plot shown in Figure 4 revealed that the Apparel line and Bais-corrected line are always closely adjacent to the Ideal line, which meant good predictive accuracy between the actual probability and predicted probability.

**Table 4:** Confusion matrix based on the testing set (*n* = 106).

		True label	
		Control group (*n* = 55)	uRSA group (*n* = 51)
Prediction	Control group, *n* (%)	40 (72.73%)	12 (23.53%)
	uRSA group, *n* (%)	15 (27.27%)	39 (76.47%)

**Figure 5:**
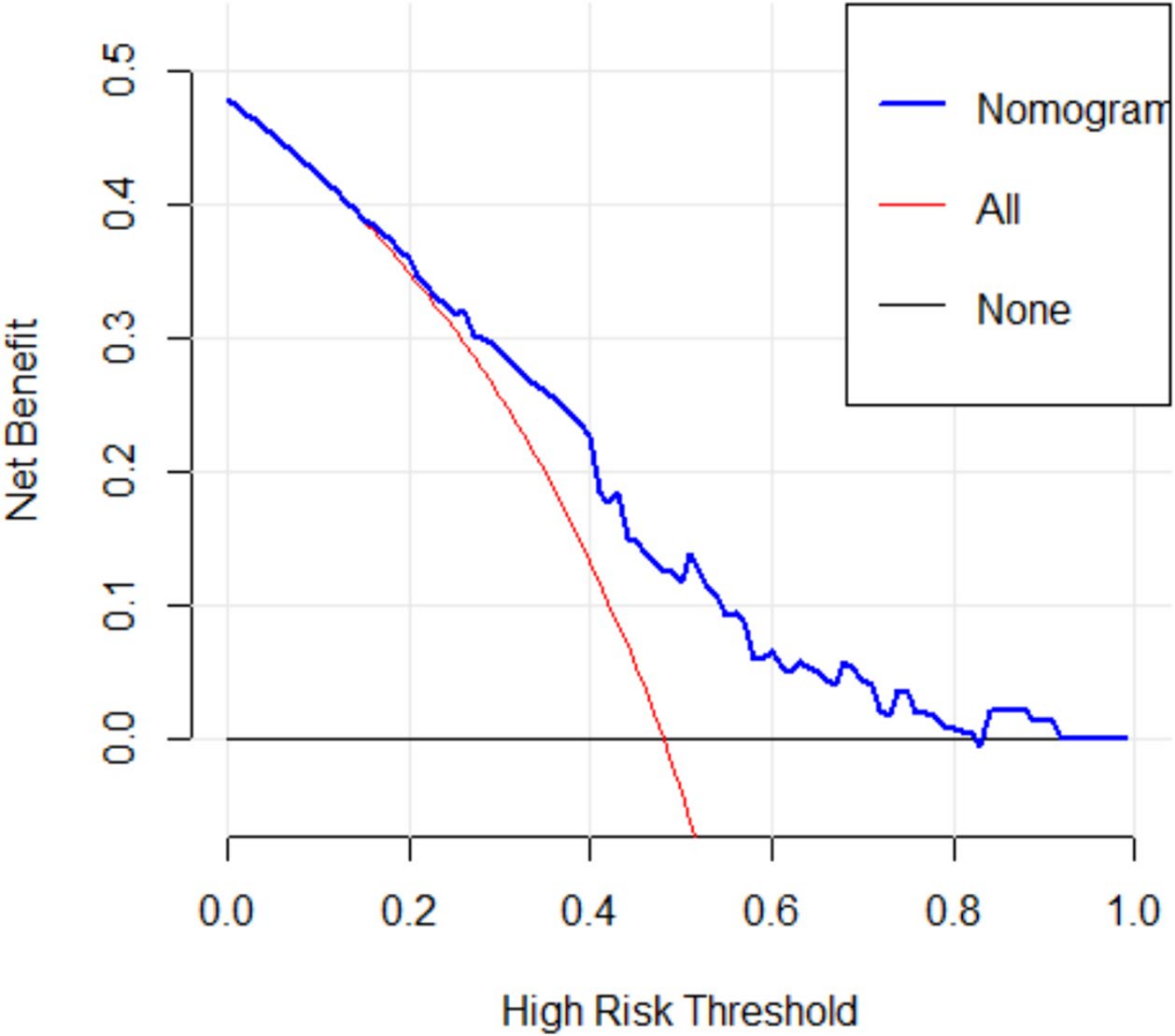
DCA decision curve of logistic regression model.

Figure 5 showed the DCA decision curve of logistic regression model. When the threshold probability was between 0.35 and 0.81, intervening on patients on the basis of the prediction model led to higher benefit than the alternative strategies of intervening on all patients or intervening on no patients. Using this model to determine whether a patient was at high risk for uRSA would improve clinical outcomes.

## Discussion

The main purpose of this study is to construct a risk prediction model for uRSA based on personal basic information and routine test items for pre-pregnancy women. According to chi-square test and clinical experience, the logistic regression model was established using the training set with FT3, FT4, TPO-Ab, TG-Ab, 25-(OH) D. The accuracy, sensitivity, and specificity of the testing set were 74.53, 72.73, and 76.47%, respectively, and the AUC of ROC curve was 0.710 (95% CI, 0.621–0.799). The calibration curve and DCA both indicated that the proposed model had good predictive performance and clinical significance for recurrent miscarriage.

In recent years, an increasing number of studies have shown that the occurrence and development of uRSA may be related to abnormal thyroid immune function and thyroid hormone disorders (13, 17, 20). TSH, FT3, and FT4 are important indicators for evaluating thyroid function. Even slight abnormalities in thyroid function during pregnancy can lead to abnormal fetal development. Previous studies have focused more on the correlation between TSH and uRSA (21). In this study, although the difference in TSH between the uRSA group and the control group was not significant, both FT3 and FT4 showed significant differences, which is crucial for the etiology analysis and prediction of uRSA.

TGAb and TPOAb are both thyroid specific antibodies (21). In this study, there was no significant difference between TGAb and TPOAb in the uRSA group and the control group. However, clinical studies have found that abnormally high level of TPOAb can lead to the occurrence of autoimmune diseases, which in turn produces autoimmune effects on fetal cells and causes miscarriage (21, 22). Abnormal elevation of TGAb level indicates abnormal thyroid follicular structure in patients, leading to increased responsiveness to the placenta and affecting the quality of the placenta and embryo, thereby increasing the risk of miscarriage (20, 22, 23). Therefore, this study also incorporated TGAb and TPOAb into the prediction model.

In recent years, many studies have shown that vitamin D not only plays a traditional role in regulating calcium and phosphorus metabolism, but also participates in physiological processes such as immunity and reproduction in the body (12, 16). Serum 25-(OH) D is the most abundant and stable vitamin D metabolite in human serum, and it is the best indicator to judge the vitamin D content synthesized by skin or ingested by food in human body (16). In recent years, studies have showed that the decline in vitamin D in pregnant women during pregnancy is likely to cause recurrent miscarriage, and may even affect fetal growth and development. Studies have shown that vitamin D can promote calcium transport in the placenta, regulate the expression of embryo implantation related genes, and participate in the establishment and maintenance of female pregnancy through vitamin D receptors (16, 24). Therefore, it can be inferred that low level of 25-(OH) D will increase the risk of spontaneous abortion (24). In this study, the 25-(OH) D level in the uRSA group were lower than those in the control group and the difference was statistically significant, consistent with relevant research conclusions.

FA is an important water-soluble vitamin, which is not only essential for the biological synthesis of deoxyribonucleic acid and ribonucleic Acid, but also a substrate for amino acid synthesis and vitamin metabolism (15, 25, 26). The metabolism of FA is closely related to deoxyribonucleic acid methylation and the formation of aneuploidy chromosomes (26). If the level of FA decreases, it can easily cause structural or numerical abnormalities in chromosomes, increasing the risk of developing uRSA (26, 27). The human body cannot synthesize folic acid on its own (15). The physiological changes of the mother during pregnancy and the growth and development of the fetus have increased the demand for folic acid (15). Serum folate is a specific index to evaluate clinical folate deficiency/deficiency (15, 25). In addition, several studies have showed the decrease of FA level can cause an increase in Hcy level, leading to abnormal endothelial and coagulation functions, inducing placental vascular thrombosis, insufficient blood perfusion, and ultimately abnormal embryonic development (15, 28). In this study, the FA level of the uRSA group was lower than that of the control group and the difference was statistically significant, consistent with the conclusions of relevant studies.

This study aimed to provide early prediction and intervention for potential patients before uRSA occurs, which is our novel contribution to the current literature. The advantage of the model proposed in this study is that it only requires routine pre-pregnancy tests to effectively indicate high-risk groups for uRSA, making it convenient for clinical application and implementation. By applying this model, clinical physicians can assess the risk of uRSA in women undergoing pre-pregnancy examinations, providing reference for further personalized examinations and early interventions. Henan Provincial People’s Hospital in this study is one of the largest comprehensive hospitals in Henan Province, and the visited patients come from various counties and cities, which ensures the generalizability of this study in Henan Province. However, it is still necessary to test the performance of the model through samples from other provinces or cities to further verify its external validity. As no specially designed survey questionnaire was distributed to the subjects, there were relatively few basic personal information characteristics included in this study. In the future, we plan to conduct a prospective cohort study incorporating more personal information and clinical test indicators, and increase the number of samples collected to further improve the prediction accuracy of the model.

## Conclusion

In this study, pre-pregnancy tests such as FT3, FT4, 25-(OH)D and FA were verified to be closely related to uRSA. TG-Ab and TPO-Ab are also important characteristics. This study successfully established a risk prediction model for uRSA based on routine pre-pregnancy tests. It is necessary to analyze the metabolomic relationship between the above tests and uRSA in the further study to improve the accuracy of uRSA risk prediction.

## Data availability statement

The original contributions presented in the study are included in the article/supplementary material, further inquiries can be directed to the corresponding authors.

## Ethics statement

The studies involving humans were approved by the Ethics Committee of Henan Provincial People’s Hospital, China (no. 2018-75). The studies were conducted in accordance with the local legislation and institutional requirements. Written informed consent to participate in this study was not required from the participants in accordance with the national legislation and the institutional requirements.

## Author contributions

JW: Writing – review & editing. DL: Data curation, Formal analysis, Writing – original draft. ZG: Investigation, Methodology, Writing – original draft. YR: Methodology, Writing – original draft. LW: Validation, Writing – original draft. YL: Formal analysis, Writing – original draft. KK: Methodology, Writing – original draft. WS: Resources, Writing – original draft. JH: Project administration, Writing – original draft. SL: Supervision, Writing – review & editing. YH: Writing – review & editing, Writing – original draft.
